# Une mucormycose mammaire compliquant un intertrigo: une localisation atypique avec évolution fatale

**DOI:** 10.11604/pamj.2014.17.5.3811

**Published:** 2014-01-13

**Authors:** Toufik Joulali, Mohammed Khatouf

**Affiliations:** 1 Service de Réanimation Polyvalente A1, CHU Hassan II, Fès, Maroc

**Keywords:** Mucormycose, intertrigo, staphylococcus doré, mucormycosis, intertrigo, staphylococcus aureus

## Image en medicine

Mme A.N, patiente de 58 ans, diabétique type 2 depuis 20 ans, ayant consulté en dermatologie pour un intertrigo sous mammaire (A), mise sous traitement avec mauvaise observance thérapeutique. La patiente est admise un mois par la suite aux urgences pour la prise en charge d'un état de choc septique avec une décompensation acéto-cétosique. L'examen Clinique a retrouvé une patiente en trouble de conscience avec un Glasgow à 8, une fréquence cardiaque à 135 bpm, une tension artérielle à 8/2, febrile à 39,5°C avec la mise en évidence d'une large nécrose cutanée surinfectée au niveau du sein droit (B). L’évolution a été rapidement fatale malgré les mesures de réanimation et une antibiothérapie à large spectre. L'examen bactériologique des prélèvements des lesions est revenu en post-mortum en faveur d'une infection à candida albicans associé à un staphylococcus doré et des éléments mycéliens d'aspect polymorphe compatibles avec des zygomycètes. Les mucormycoses sont des infections fongiques opportunistes rares mais graves, survenant chez les diabétiques et les patients immuno-déprimés. La localisation rhino-orbito-cérébrale est la forme la plus fréquente représentant 40 à 50% des cas. Le diagnostic repose sur l'examen mycologique et anatomo-pathologique et le taux de mortalité reste assez élevé à cause des erreurs diagnostiques et du retard de la prise en charge.

**Figure 1:**
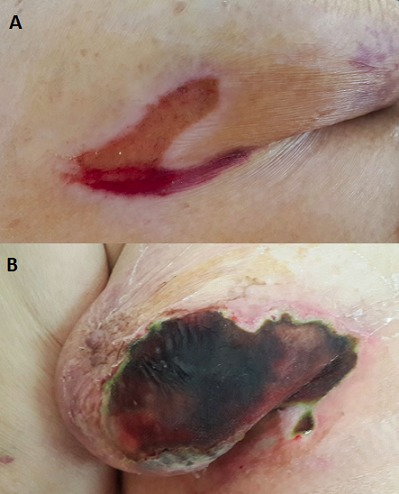
A) Image des lésions initiales: intertrigo sous mammaire; B) Image de la nécrose cutanée en rapport avec une mucormycose

